# Activation of H^+^-ATPase of the Plasma Membrane of *Saccharomyces cerevisiae* by Glucose: The Role of Sphingolipid and Lateral Enzyme Mobility

**DOI:** 10.1371/journal.pone.0030966

**Published:** 2012-02-16

**Authors:** Sergey Permyakov, Nataliya Suzina, Airat Valiakhmetov

**Affiliations:** 1 Institute for Biological Instrumentation, Russian Academy of Sciences, Pushchino, Moscow Region, Russia; 2 Skryabin Institute of Biochemistry and Physiology of Microorganisms, Russian Academy of Sciences, Pushchino, Moscow Region, Russia; University of Cambridge, United Kingdom

## Abstract

Activation of the plasma membrane H^+^-ATPase of the yeast *Saccharomyces cerevisiae* by glucose is a complex process that has not yet been completely elucidated. This study aimed to shed light on the role of lipids and the lateral mobility of the enzyme complex during its activation by glucose. The significance of H^+^-ATPase oligomerization for the activation of H^+^-ATPase by glucose was shown using the strains *lcb1-100* and *erg6*, with the disturbed synthesis of sphyngolipid and ergosterol, respectively. Experiments with GFP-fused H^+^-ATPase showed a decrease in fluorescence anisotropy during the course of glucose activation, suggesting structural reorganization of the molecular domains. An immunogold assay showed that the incubation with glucose results in the spatial redistribution of ATPase complexes in the plasma membrane. The data suggest that (1) to be activated by glucose, H^+^-ATPase is supposed to be in an oligomeric state, and (2) glucose activation is accompanied by the spatial movements of H^+^-ATPase clusters in the PM.

## Introduction

H^+^-ATPase, hereafter, Pma1, of the yeast plasma membrane (PM), one of the major structural proteins of the PM, belongs to a P-type ATPase [1], [2] and is encoded by the PMA1 gene [3]. The enzyme hydrolyzes ATP and forms an electrochemical proton gradient on the PM and drives the transport of basic nutrients across the PM [4]. As has been shown previously, Pma1 consumes up to 20% of cellular ATP and, not surprisingly, its activity is under strict control [5]. The regulation of Pma1 activity was shown for the first time by Serrano, who discovered that incubation with glucose resulted in a reversible many-fold enhancement of the enzyme's activity, a decrease in K_m_, and an increase in V_max_ [6]. The sugars utilized via the glycolytic pathway (fructose and mannose) were shown to lead to the enhancement of the enzyme's activity [6]. Sugars (D-xylose and galactose) metabolized through other pathways, as well as nonmetabolized glucose analogs (3-O-methylglucose and deoxyglucose), did not result in any enhancement.

Quite recently, it has been shown that glucose-dependent Pma1 activation is accompanied by double phosphorylation of the enzyme at the Ser-911 and Thr-912 positions [7]. Phosphorylation of these two residues is thought to eliminate the inhibitory effect of the enzyme's C-terminus on the ATP-hydrolyzing domain [7]–[9]. It is possible that the process of Pma1 phosphorylation involves protein kinase C, the activation of which is associated with the transport and phosphorylation of glucose [10].

Electron cryomicroscopy and crystallographic analysis were used previously to determine the tertiary and quaternary structure of the enzyme [11], [12]. Pma1 was shown to form detergent-resistant hexamers [13], [14], within which the monomers contact each other at the levels of both the transmembrane and cytoplasmic domains [12]. Another important finding was that the hexameric structure has internal mobility and becomes more closely packed under substrate binding [15].

As has been shown, some membrane proteins have to be associated with lipid rafts for correct incorporation into the plasma membrane [16]. To be delivered to and incorporated into the plasma membrane [17]–[20], Pma1 must also form a complex with sphingolipid. In the cells with disturbed sphingolipid synthesis, the newly synthesized native Pma1 has been shown to be routed, instead of to the PM, to the vacuole where it is degraded [17], [21]. Previous experiments with PMA1-GFP have allowed direct visualization of the raft compartmentalization of Pma1 [22].

A recently proposed model of membrane organization has suggested that all membrane proteins are contained both in the rafts and in the nonraft lipid domains and separated by vast protein-free lipid regions [23]. All protein-lipid “islands” are also thought to be bound to cytoskeleton elements [23]. This idea has aroused particular interest due to the finding of an acetylated tubulin-Pma1 complex in glucose-starved yeast and its dissociation under glucose addition [24]. Research has shown that the acetylated tubulin-Pma1 complex is dissociated very rapidly and that the glucose-induced increase in Pma1 activity occurs after its disintegration [24]. On the other hand, in *S. cerevisiae*, the formation and stability of Pma1-containing patches have been shown not to depend on the integrity of the actin and tubulin structures [25]. It has also been demonstrated that Pma1 molecules are relatively mobile within these patches [25].

The study of lateral mobility and oligomerization of transmembrane proteins has been based mainly on the phenomenon of fluorescence resonance energy transfer (FRET) [26]–[28]. When the donor and the acceptor carry different fluorophores, the distance between them can be assessed by changes in the fluorescence emission spectrum. If the donor and acceptor molecules carry the same fluorophore, then the intermolecular interactions can be studied by the change in fluorescence anisotropy [28], [29]. The latter method has been denoted as homo-FRET and has been widely used recently to estimate the degree of protein oligomerization [28].

Thus, it is clear that glucose activation of Pma1 is a complex process including several levels. In this work, we have attempted to assess (a) the role of sphingolipid and ergosterol in the glucose activation of Pma1 and (b) the mobility of yeast Pma1 molecules under glucose-induced activation of the enzyme.

## Materials and Methods

### Strains and growth conditions

*S. cerevisiae* strains (Table 1) were grown on standard YPD medium (Sigma, USA) for 16–18 h (late exponential phase) and were twice washed with water and incubated in distilled water for 1 h at 28°C to completely eliminate the effect of glucose from the growth medium. The strains *erg-6*, *lcb1-100*, *SEY6210*, and *GFP-PMA1* were a kind gift from Dr. W. Tanner (University of Regensburg, FRG); the strain *BY4742* was a kind gift from Dr. J. Boek (Johns Hopkins University, Baltimore, USA); and the strain *RH2874* was a kind gift from Dr. H. Riezman (University of Geneva, Switzerland).

**Table 1:** Yeast strains used in this study.

Strain	Genotype	Reference
SEY6210	MATα ura3-52 leu2-3,112 his3-Δ100 trp1-Δ901 lys2-801suc2-Δ9	[30]
PMA1-GFP	SEY6210 except PMA1: :GFP: : kanMX4	[22]
BY4742	MATα his3-Δ1 leu2-Δ0 lys2-Δ0 ura3-Δ0 trp1-Δ901 bar1-1	[31]
erg6	BY4742 except erg6Δ: :kanMX4	[22]
RH2874	MATα leu2 ura3 lys2 trp1 bar1-1	[32]
lcb1-100	MATα lcb1-100 leu2 trp1 ura3 lys2 bar1-1	[32]

### Incubation with sugars and cell permeabilization

The cells were resuspended in permeabilization buffer (PB) (0.5 M sorbitol, 100 mM KCl, 4 mM MgCl_2_, 10 mM MES, pH 6.5) at a concentration of 1 g/10 ml and 5 ml samples were poured into 15 ml test tubes. Then, 0.25 ml of 2 M glucose (deoxyglucose) (Sigma, USA) was added to the experimental samples, and 0.25 ml of water was added to the controls. The samples were incubated for 15 min at 28°C. Then, 0.25 ml of 5% Triton X-100 was added to all of the samples. Immediately after, the samples were frozen at −60°C in a deep freezer and left overnight.

### Determination of Pma1 activity in situ

The permeabilized cells were thawed at 28°C, washed 3 times with 5 ml of PB, resuspended in 2 ml of the same buffer, and stored at 0°C until use. A total of 20 µl of 100 mM Mg-ATP (Boehringer, USA), 1 µl of 10 µM Bafilomycin (Sigma, USA) in DMSO (for inhibition of the vacuolar ATPase), and 50 µl of cell suspension were added to 910 µl of PB. Then, 20 µl of 5 mM VO_4_ was added to the controls for the inhibition of Pma1. The reaction mixture was incubated at 30°C at 1,000 rpm in a Thermomixer compact (Eppendorf, FRG) for 30 min. The cells were precipitated at 14,000 g for 3 min, and the aliquots were diluted 50–100 times with ddH_2_O. In a 96-well microplate, 200 µl of malachite green (Applechem, FRG) was added to 100 µl of a sample [33] and incubated at 30°C for 15 min; the content of released P_i_ was measured at 650 nm. Pma1 activity was calculated by the difference in the amount of inorganic P_i_ released in the presence and absence of 100 µM sodium orthovanadate, the specific inhibitor of Pma1 [9]. To calculate the specific enzyme activity, total cell protein was determined [34] with modifications. A total of 100 µl of the cell suspension in the PB was added to 1 ml of water and precipitated at 14,000 g. The precipitate was resuspended in 0.6 ml of water and 0.3 ml of 3 N NaOH was added and heated at 100°C for 5 min, followed by addition of 0.3 ml of 2.5% CuSO_4_ after cooling. Five minutes later, the mixture was centrifuged at 14,000 g, and the supernatant was measured by spectrophotometry at 555 nm. Specific Pma1 activity was expressed as nmol of inorganic P_i_ released from ATP per minute per mg of total cell protein.

### Plasma Membrane Isolation and ATP Hydrolysis

The plasma membranes were prepared from the glucose-metabolizing yeast cells by centrifugation on a sucrose step-gradient as previously described [35]. The Pma1 assays were conducted in 96-well microplates, essentially as described [36]. The basic ATP hydrolysis assay medium consisted of 5 mM MgSO_4_, 4 mM ATP, 25 mM NH_4_Cl, and 10 mM MES-Tris, pH 6.5. The reaction was performed in 150 µl of volume with 1.0 µg of membrane protein at 30°C for 20 min. The reaction was stopped by the addition of 100 µl of a combined Stop-Color Development reagent containing 1% SDS, 0.8% ascorbic acid, 100 mM ammonium molybdate, and 0.6 M H_2_SO_4_. Pma1 activity was calculated by the difference in inorganic P_i_ released from ATP in the absence and presence of 10 µM sodium orthovanadate [35]. The K_m_ was calculated using Prism software (GraphPad Software Inc., La Jolla, CA).

### Determination of anisotropy

Steady-state fluorescence anisotropy measurements were performed using a Cary Eclipse spectrofluorimeter (Varian Inc. USA) equipped with an automated polarizer accessory. The cell holder temperature was kept at 28°C. An aqueous cell suspension with an absorbance about 0.3 at 600 nm was used. GFP fluorescence was excited at 480 nm, and the emission was collected at 520 nm. The excitation bandwidth was 10–20 nm, and the emission bandwidth was 20 nm. Appropriate blanks were measured using GFP-lacking cells. The difference between the cell concentrations of the main and the reference solutions was estimated from their absorbance at 600 nm. Each contribution of the reference solution was corrected for this difference using the Beer-Lambert law [37]. The blanks were subtracted from each of the fluorescence intensity values (I_VV_, I_VH_) and used to calculate the anisotropy values [38]. All of the fluorescence anisotropy values were corrected for the instrumental G factor, which was measured using a highly diluted aqueous solution of fluorescein. The values reported represent an average of three measurements with an average time of 10 s.

### Immunogold assay

The cells were fixed with 1.25% glutaraldehyde-1% paraformaldehyde in 0.1 M cacodylate buffer (pH 7.0). The samples were washed with the buffer, dehydrated in ethanol, infiltrated with LR white, omitting acceleration, and cured for 40 h at 50°C. The ultrathin sections obtained were mounted on copper grids covered with a formvar support. This was followed by incubation with polyclonal anti-Pma1 antibodies (a kind gift from Dr. C. Slayman, Yale University, USA), diluted (1∶100) in PBS for 2 h at 30°C, and again washed 2 times for 5 min in PBS. The grids were incubated with protein A (Serva, FRG) conjugated with 15-nm-diameter gold particles for 1 h at 30°C and washed 2 times for 5 min in PBS and finally once in water. After air drying, the samples were stained with a 3% uranyl acetate solution in 70% ethanol for 30 min and washed 2 times for 5 min in PBS. The sections were examined in a JEM100B (JEOL, Japan) electron microscope at an accelerating voltage of 80 kV.

### Results and Discussion

The association of Pma1 with lipid rafts has been studied quite thoroughly [16]–[21], [39]. However, little is known about the role of lipids in glucose activation of Pma1.

To shed light on this issue, we selected the mutant *erg6*, which is deficient in ergosterol, and the mutant *lcb1-100*, which is defective in sphingoid base synthesis. The incubation of prestarved cells with 100 mM glucose or 100 mM deoxyglucose for 15 min yielded the results shown in Table 2. As expected, both of the parent strains, *BY4742* and *RH2874*, demonstrated a glucose effect with different degrees of intensity. Unsurprisingly, the *erg6* strain also showed glucose activation comparable with the parent strain. Previously, it was reported that disturbances in ergosterol synthesis in no way affected Pma1 biogenesis, secretion on the PM, or stability [19]. In addition, ergosterol was shown to occur mainly in the membrane patches containing arginine and uracyl symporters but not Pma1 [40].

**Table 2:** Pma1 activity in situ (nmol P_i_/min/mg total cell protein, *n* = 3±SD) after 15-min incubation of *S. cerevisiae* whole cells with 100 mM glucose or 100 mM deoxyglucose. The change of activity in % of initial activity is given in parenthesis.

Strain	Buffer	100 mM glucose	100 mM deoxyglucose
BY 4742	7.36±2.94 (100)	14.64±1.16 (198.94)	5.64±1.54 (76.6)
erg6	22.47±0.35 (100)	44.0±0.1 (195.85)	24.8±0.35 (110.38)
RH 2874	29.62±0.75 (100)	38.68±1.74 (130.59)	21.27±0.36 (71.81)
lcb1-100	38.34±0.96 (100)	35.73±2.54 (93.2)	36.18±0.13 (94.35)
SEY 6210	18.5±0.89 (100)	52.36±0.85 (283.1)	16.8±2.19 (91)
PMA1-GFP	7.27±1.68 (100)	23.19±0.22 (319.1)	5.59±0.98 (77)

In contrast, in strain *lcb1-100*, incubation with glucose did not increase the enzyme activity and even slightly reduced it. At the same time, the basal Pma1 activity in strain *lcb1-100* was somewhat higher than in the parent strain *RH2874*. The latter result was quite unexpected, because previously it had been shown for the *lcb1-100* strain that only a minor part of the newly synthesized Pma1 reaches PM at 30°C, whereas more than 90% of the enzyme is rerouted to the vacuole to be degraded [17]. However, later work suggested that this effect depended in many respects on the cultivation medium [20]. It should be noted that the growth of the *lcb1-100* strain on the YPD medium was almost twice as low as that of the parent strain *RH2874* (data not shown). With the exception of the *erg6 s*train, deoxyglucose did not result in an increase in the Pma1 activity, in agreement with the Serrano's data [6].

Thus, we have shown that sphingolipid but not ergosterol is important for glucose activation of Pma1. This fact can be explained as follows: One of the consequences of sphingolipid synthesis disturbance in the *lcb1-100* strain is inefficient or completely blocked Pma1 oligomerization [17], [18], [20], which probably results in the elimination of glucose activation. The difference in glucose effects on Pma1 activity in the *erg6* and *lcb1-100* strains may, therefore, be attributed to the sphingolipid associating with the protein at the very initial stages of biosynthesis of the enzyme and determining its oligomeric structure [18], [20]. Ergosterol, the other component of the lipid raft, appears not to participate directly in the formation of the oligomeric Pma1 complex and have no particular effect on the functioning of the protein. The idea that oligomerization of Pma1 is necessary for the glucose activation of Pma1 was indirectly confirmed in the earlier work [12]. Using electron crystallography, researchers showed that the cytoplasmic part of Pma1 in a ligand-free form consists of four domains [12]. Domain two of one Pma1 molecule directly contacts domain three of the neighboring molecule. Unfortunately, the authors of this work did not link these structural domains with the functional (ATP-binding, phosphorylation, C-terminal) domains. However, it may be hypothesized that in the absence of glucose, the nucleotide-binding domain of the Pma1 molecule is locked by the C-domain of the neighboring Pma1 molecule. In this case, glucose activation of the enzyme results in successive phosphorylation of Ser-911 and Thr-912, followed by the release of the C-tail from the nucleotide-binding domain, as demonstrated previously [7], [9]. Taking into account the intermolecular character of the described event, it may be supposed that Pma1 oligomerization is necessary for the activation of Pma1 by glucose.

Since the modern concept of glucose activation of Pma1 presupposes the movement of its C-tail [7], [9], this process could be traced using the strain *PMA1-GFP*, the Pma1 molecule of which carries a GFP domain at the C-terminus [22]. To elucidate the effect of this spectral marker on glucose activation of the enzyme, the influence of the 15-min incubation of whole cells with 100 mM glucose or 100 mM deoxyglucose on Pma1 activity was investigated (Table 2). Both the parent strain *SEY6210* and the strain *PMA1-GFP* demonstrated a marked glucose effect that exceeded that of the *BY4742* and *RH2874* strains. Although the presence of the GFP domain resulted in a considerable decrease in basal Pma1 activity (18.5 and 7.3 nmol P_i_/min/mg total cell protein for *SEY6210* and *PMA1-GFP*, respectively), it had little effect on the K_m_ of the glucose-activated enzyme. The K_m_ values of Pma1 determined in the membrane fraction from glucose-activated cells of the *SEY6210* and *PMA1-GFP* strains were 0.22 mM and 0.41 mM, respectively. It is known that the K_m_ for glucose-activated Pma1 is 0.3–0.8 mM, in contrast to 2–4 mM for Pma1 in glucose-deprived cells [6], [36]. Thus, the *PMA1-GFP* strain affords an opportunity to register the glucose activation process using modern spectral methods.

Fluorescence anisotropy was used to observe the structural rearrangements in the Pma1 molecules. The value of fluorescence anisotropy ***r*** for the GFP monomer in the absence of substantial rotation of the fluorophore molecule during the fluorescence lifetime is close to the maximum value (0.4) [41]–[43]. The radiationless transfer of energy between the fluorophore molecules (homo-FRET) as a result of oligomerization of the GFP molecules has been shown to result in a decrease in the observed effective value ***r*** [44]. Table 3 shows the ***r*** values of *PMA1-GFP* whole cells in the absence and presence of (deoxy)glucose. Since the procedure for measuring the value of ***r*** involves subtraction of the contribution of cellular autofluorescence to the total fluorescence, the observed ***r*** value corresponds to that of the GFP molecules. As can be seen, the experimental ***r*** values (0.165–0.202) were much lower than 0.38, the value ***r*** for the monomeric and dimeric form of GFP expressed in different samples [44]. The decrease in ***r*** was typical of the homo-FRET phenomenon observed for the clusters of fluorophores approaching each other until the distance between them was less than 10 nm.

**Table 3:** Fluorescence depolarization (anisotropy) **r** of PMA1-GFP in whole cells after 15 min incubation with 100 mM glucose or deoxyglucose.

Additions	r
None	0.192±0.005
100 mM glucose	0.165±0.005
100 mM deoxyglucose	0.202±0.003

Since it has been shown previously that the supramolecular Pma1complex in the PM consists of six units [11]–[13], the value ***r*** in the absence of (deoxy)glucose (equal to 0.192) characterizes the anisotropy of the PMA1-GFP hexameric complex. The incubation of the *PMA1-GFP* cells with 100 mM glucose for 15 min resulted in a marked decrease in the ***r*** value by 0.026±0.005. The same incubation with deoxyglucose produced an insignificant opposite effect: an increase in the ***r*** value by 0.011±0.006. Thus, there is a correlation between the values of Pma1 activity (Table 2) and fluorescence anisotropy (Table 3). This finding implies that glucose activation of the enzyme is accompanied by certain structural rearrangements of the Pma1 molecules at the level of clusters. The following variants and their combinations are possible [44], [45]: a change in the number of PMA1-GFP molecules in a cluster or association of clusters with each other, a change in the average distance between the Pma1 molecules in a cluster, or regrouping/reorientation of the GFP domains without a change in the distance between the Pma1 molecules. The most probable explanation for the decrease in the ***r*** value in our study seems to that since the activated enzyme intensively hydrolyzed ATP, the enzyme remained in the ligand-bound conformation for some of the time. Previously, it has been shown that the cytoplasmic Pma1 domain is more closely packed in this conformation [15]. It has also been proposed that phosphorylation of Ser-911 and Thr-912 during glucose activation results in conformational changes in the Pma1 molecule in which the C-domain is released from the nucleotide-binding domain [7], [9]. In our model system, the C-tail of Pma1 carried a GFP domain. The decrease in the ***r*** value may point to approaching of GFP domains within a hexamer complex during glucose activation of Pma1. However, in the case of a high fluorophore concentration, which often occurs in the case of membrane-bound proteins, the phenomenon of homo-FRET theoretically may be not a consequence of fluorophore oligomerization. Instead, it may be the result of the close approach of monomeric proteins (the so-called concentration depolarization effect) [28].

**Figure 1:**
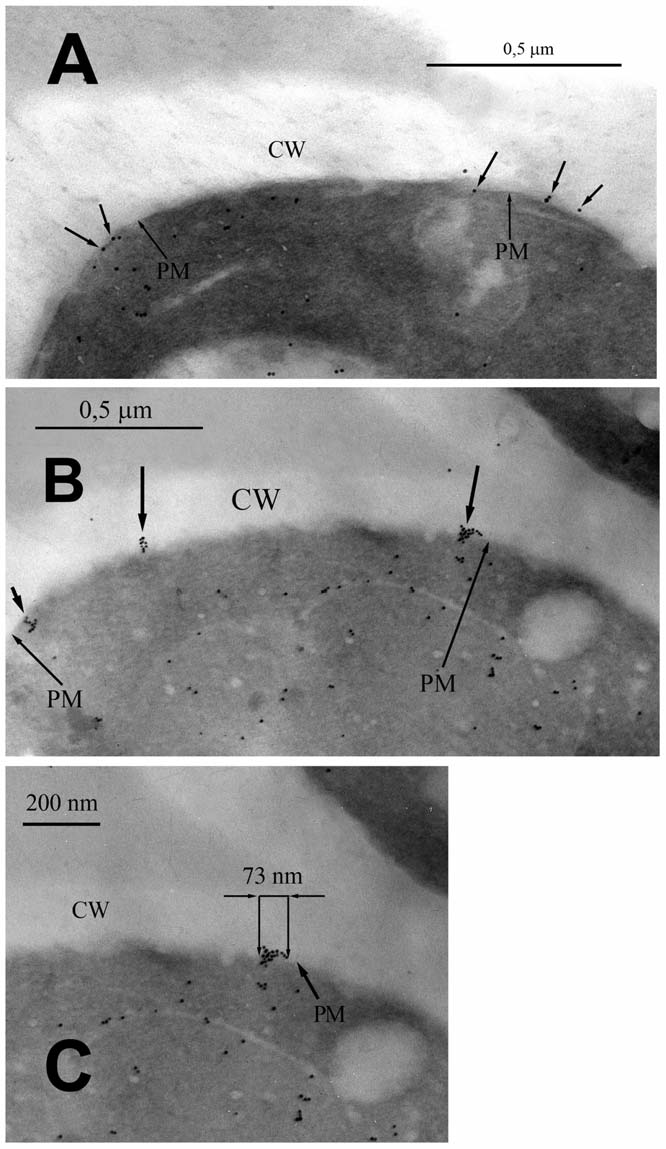
Immunogold labeling of Pma1 in the plasma membrane of *S. cerevisiae SEY6210*. (A) – glucose-starved cells, Pma1 was distributed in the membrane as single structures; (B) – cells that had metabolized glucose for 15 min, Pma1 formed large bunch-like complexes; (C) – enlarged fragment of photograph (B) CW = cell wall; PM = plasma membrane.

The change in the spatial distribution of the clusters containing Pma1 molecules during glucose metabolism was visualized using immunogold labeling. As Figure 1 shows, the distribution of the clusters of Pma1 molecules in the PM underwent substantial changes. In the glucose-starved cells of the parent strain *SEY6210*, the distribution of the clusters of Pma1 molecules was relatively uniform, and the average distance between them exceeded 10 nm (Fig. 1A). In the cells that had metabolized glucose, the clusters of Pma1 molecules aggregated in large groups of closely adjacent clusters (Fig. 1B). Since immunogold labeling was performed on a cell cross-section but not on the inner leaflet of the PM obtained by freeze-fractioning, the size of the groups cannot be analyzed using Ripley's K-function. It is interesting that the approximate sizes of these groups of clusters of Pma1 molecules (about 70 nm) (Fig. 1C) are similar to the sizes of the individual lipid rafts (25–70 nm) [46], [47]. The distance between the Pma1 clusters in such a group was comparable to the distances at which the homo-FRET effect was exhibited (10 nm); therefore, the observed decrease in fluorescence anisotropy in response to glucose addition can be at least partially explained by this phenomenon. While intracellular labeling can be observed in all of the images, we assume that it is the labeling of the newly synthesized Pma1 molecules during their delivery to PM.

**Figure 2:**
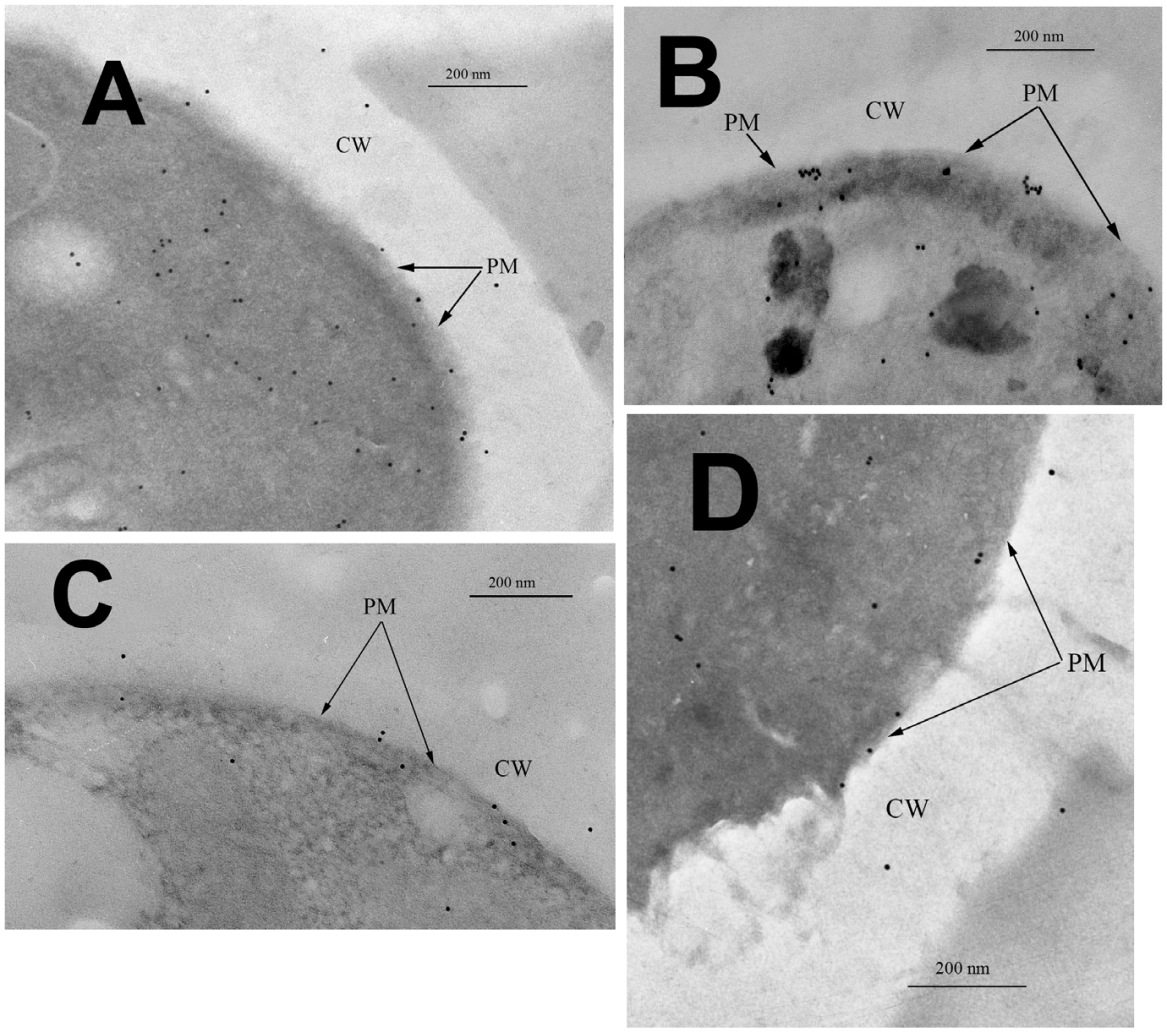
Immunogold labeling of Pma1 in the plasma membrane of *S. cerevisiae erg6* and *lcb1-100*. (A) – glucose-starved cells of the *erg6* strain, Pma1 was distributed in the membrane as single structures; (B) – *erg6* cells that had metabolized glucose for 15 min, Pma1 formed complexes; (C) - glucose-starved cells of the *lcb1-100* strain, Pma1 was distributed in the membrane as single structures; (D) – *lcb1-100* cells that had metabolized glucose for 15 min, Pma1 was distributed in the membrane as single structures. CW = cell wall; PM = plasma membrane.

Figure 2 shows the effect of incubation with glucose on the spatial distribution of the Pma1 molecules in the PM of the *erg6* and *lcb1-100* strains. In the *erg6* strain (Fig. 2A, B), where glucose activated Pma1, the distribution of the clusters of enzyme molecules before and after the incubation with glucose was similar to the distribution of Pma1 in the *SEY6210* strain. In the absence of glucose, Pma1 in the PM was distributed uniformly as separate particles. Following the incubation with glucose, Pma1 formed groups. In contrast to the *SEY6210* and *erg6* strains, the incubation with glucose did not change the distribution of Pma1 in the PM of the *lcb1-100* strain (Fig. 2C, D). The distribution of the Pma1 clusters remained uniform, without the formation of groups. Moreover, glucose did not exert an effect on the *lcb1-100* strain (Table 2). Thus, it may be supposed that Pma1 activation by glucose is accompanied by the formation of groups of Pma1 clusters.

The revealed glucose-dependent movement of the clusters of the Pma1 molecules is noteworthy because it is a rather quick process (about 15 min). It is unclear what cellular mechanism underlies this reorganization of the Pma1 clusters, but one of the most probable reasons is the previously reported association of Pma1 with acetylated tubulin [24]. In this study, the authors showed that Pma1 in the cells that did not metabolize glucose was associated with acetylated tubulin. On glucose addition, this complex very quickly degraded. These findings, together with our data presented above, suggest that acetylated tubulin in the absence of glucose fixes Pma1 clusters on the membrane and leads to a uniform distribution. Upon glucose addition, this complex disintegrates, allowing the clusters of Pma1 molecules to move freely in the membrane plane and combine into large groups.

These findings lead to the following two conclusions: (1) for glucose activation of Pma1 to take place, the enzyme is supposed to be in the oligomeric state, and (2) glucose activation is accompanied by the spatial movement of Pma1 clusters in the PM.
